# *Helcococcus ovis* associated with septic arthritis and bursitis in calves – a case report

**DOI:** 10.1186/s12917-021-02996-6

**Published:** 2021-09-03

**Authors:** Alexandra Jost, Marlene Sickinger

**Affiliations:** Clinic for Ruminants (Internal Medicine and Surgery), Department of Veterinary Medicine, Justus-Liebig-University of Giessen, Frankfurter Str. 104/106, 35392 Giessen, Germany

**Keywords:** Calves, Herd health, Arthritis, *Helcococcus ovis*

## Abstract

**Background:**

Septic arthritis often occurs in young calves when the passive transfer of maternal immunoglobulins has failed, which results in hypogammaglobulinaemia in the calf. Another important cause is suboptimal herd health management which often leads to general health impairment and, subsequently, to septic arthritis.

**Case presentation:**

A dairy farmer consulted the Herd Health Service of the University Clinic reporting general herd health impairment, a high incidence of respiratory diseases, unsatisfactory weight gain and arthritis in calves, as well as mastitis and high milk cell counts. Clinical examinations were performed, and diagnostic measures were taken. A transtracheal lavage (TTL) was performed, and synovial swab samples were taken from the carpal joint and the subcutaneous tarsal bursae of two calves. Microbiological examinations of synovial swabs revealed co-infections of *Trueperella pyogenes* and *Helcococcus ovis* in one calf and *Helcococcus ovis* in pure culture in the other. The TTLs confirmed the presence of *Mycoplasma spp.* associated with respiratory diseases.

**Conclusions:**

*Helcococcus ovis* is currently regarded as a co-infective bacterial agent. However, it seems to play a significant role as the primary pathogen in this case.

## Background

Pneumonia and septic arthritis with occasionally associated tenosynovitis are two of the most common diseases and causes of mortality in dairy, beef and crossbred calves raised for the production of white veal [1, 2]. The economic losses of these diseases result mainly from unsatisfactory weight gains in calves and feedlot cattle as well as reduced milk yield and impaired fertility in dairy cows. The aforementioned clinical symptoms in calves and dairy cows are direct effects of arthritis-related lameness in affected animals [3, 4]. Septic arthritis and inflammation of the synovial structures are mainly caused by trauma, expansion of periarticular inflammation or the haematogenous spread of bacteria [5–10]. Bacteria commonly associated with septic arthritis in cattle are *Trueperella pyogenes*, *Streptococcus spp.*, *Staphylococcus spp.* and *E. coli* [11, 12]. The latter has been used to induce septic arthritis in Holstein calves for experimental purposes [13]. Haematogenous spreading and the direct link between pneumonia and arthritis in animals with bovine respiratory disease (BRD) are well-researched features [14] and have often been associated with *Mycoplasma spp.* [15, 16]. However, within the last years, *Helcococcus ovis* has been discussed as a newly emerging pathogen associated with pulmonary diseases and several other pathologic conditions [17–19]. This pathogen has been diagnosed as the causative agent of septic arthritis in calves at a dairy farm in the mid-west of Germany. To date, *Helcococcus ovis* was mostly found in combination with other pathogenic agents [20]. The fact that it was possible to isolate this bacterium from septic arthritis and bursitis makes for a particularly interesting finding.

## Case presentation

A local dairy farmer requested a consultation with the Herd Health Service of the Clinic for Ruminants (Internal Medicine and Surgery). The reasons included respiratory problems and cases of purulent arthritis in the calves. During the consultation, he also reported impaired fertility with high rates of retained fetal membranes and cases of mastitis in the dairy cows. Because calves’ issues were considered separate from those of the dairy cows, a second consultation was advised. Thus, the possible reasons dairy cows’ impaired health status will not be discussed in this manuscript.

During the herd health consultation, the farmer presented several calves with pneumonia and swollen carpal and tarsal joints.

A sample of two calves was drawn, and they were examined more closely. They were Holstein Friesian calves, of the ages two (calf 1, male) and four (calf 2, female) months. Both calves showed a poor nutritional status with a long, scruffy coat. The estimated body weights were 70 and 120 kg, respectively. The vital parameters were within the reference ranges (heart frequency: 72–92 bpm; respiratory frequency: 20–40 per min; body temperature: 38.5–39.2 °C; [21]), but the symptoms of BRD were pronounced, including coughing as well as inspiratory and expiratory snarling and rattling. The examination of the musculoskeletal system revealed moderate to severe lameness in both calves. The animals showed mixed lameness in the right hind leg (calf 1: degree 2–3/5) and the right front leg (calf 2: degree 4–5/5).

**Fig. 1:**
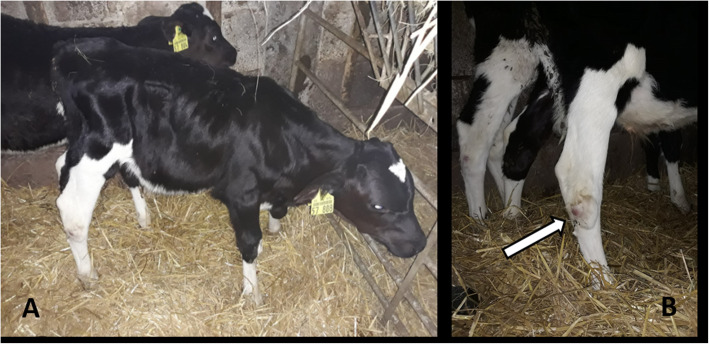
**A** Two-month-old male calf with chronic inflammation in the lateral tarsal bursa of the right hind leg. **B** The calf’s severe tarsal bursa swelling is indicated by an arrow

The orthopaedic examination in calf 1 (2 months old, male) indicated decubitus in both carpal joints and marked swelling of the lateral tarsal bursa on the right hind leg (Fig. 1). Medially, the skin covering the tarsal joint revealed a superficial lesion. Additionally, this calf showed severe 3 × 2 × 1 cm-sized swelling of the subcutis and buccal mucosa of the right mandible. Palpation of the mandibular bone did not yield any pathological findings. It was assumed that the multiple symptoms displayed by this calf (BRD, inflammatory swelling of the buccal mucosa and bursitis) were caused by the haematogenous spread of bacteria rather than a local infection related to the medially located, superficial skin lesion.

A sterile puncture of the lateral tarsal bursa was performed. Macroscopically, the bursal synovial liquid was slightly yellowish and of medium turbidity. The viscosity was slightly reduced immediately after puncture but developed a slightly jelly-like consistency within approximately 20 min after sampling. The farmer was presented with the diagnosis of serofibrinous tarsal bursitis in calf 1 and treatment options (transfer to the clinic and bursal lavages with consecutive antibiotic treatment) were discussed. The owner decided to have his calf treated on the farm by the local veterinarian. An antibiotic treatment containing Procaine-Penicillin (30.000 IU/kg once daily, i.m.) was prescribed. In a retrospective phone call, the farmer reported that the calf had received Procaine-Penicillin for 7 days and had recovered without needing bursal lavages.

The clinical and orthopaedic examination of calf 2 (4 months old, female) revealed a stiffening of the right carpal joint due to chronic septic arthritis. It was not possible to extend the calf’s right carpal joint flexure passively.

**Fig. 2:**
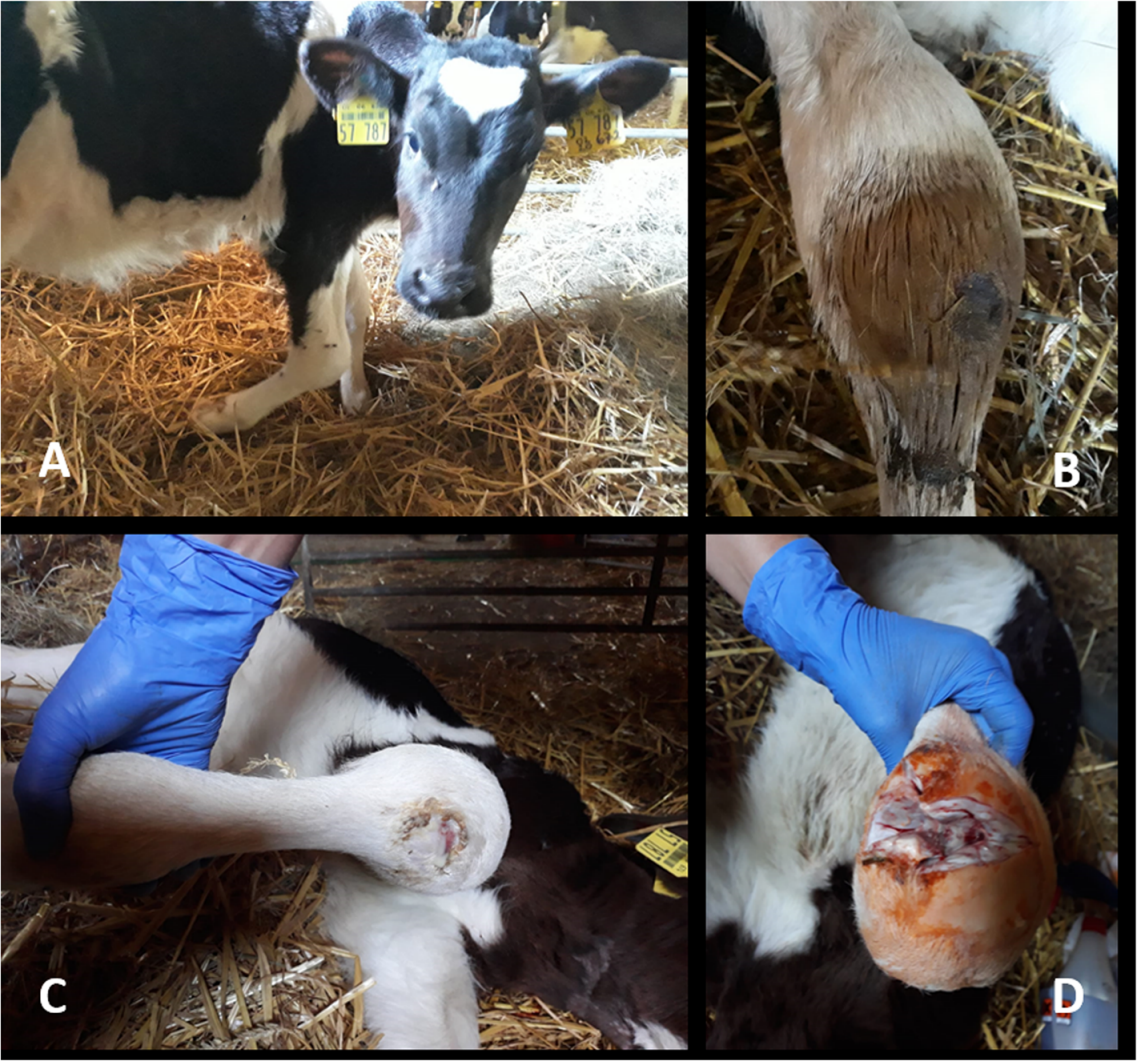
**A** A 4-month-old female calf suffering from arthrogryposis due to chronic septic arthritis. **B** The calf’s subcutaneous carpal bursa with an encrusted area in a dorso-medial position along the carpus. **C** Removal of the encrusted material after euthanasia revealed purulent secretion. **D** Preparation of the carpal joint for swab sampling

The calf was not able to move physiologically and showed a lameness degree of 4–5/5. The carpal joint circumference was extremely swollen and, after removing the encrusted tissue, the dorsal area of the subcutaneous carpal bursa oozed a purulent substance (Fig. 2).The owner was advised to transfer this second calf to the clinic for further diagnostics including radiography and ultrasonography. However, he declined due to financial limitations. Thus, it was not possible to perform an ultrasound or x-rays. Because further intravital diagnostics were not authorised, the owner was unable to pay for surgical treatment (arthrotomy), and it was impossible to restore the carpal joint conservatively (antibiotic treatment and joint lavage); the animal had to be euthanised. The post-mortem findings revealed fibrinous material within the joint capsule, and the periarticular soft tissue consisted of connective tissue with a greasy, whitish surface. The diagnosis stated: chronic, fibrinous carpitis with periarticular soft tissue inflammation. In cases of chronic fibrinous carpitis, dorsal longitudinal ultrasonography (4.5–18 MHz linear transducer) is indicated. The intercarpal joint space and a severely distended joint recess with inhomogenous, hyperechogenic material (fibrin) within the joint capsule may be displayed using this technique.

Within the scope of diagnostic measures, blood samples, TTLs and a bursal synovial sample (calf 1, lateral tarsal bursa) and synovial swabs were taken. The synovial samples could not be examined cytologically because the synovial liquid had clotted upon arrival at the clinic. Microbiological examinations and PCR diagnostics were performed to prove the possible involvement of *Mycoplasma species*. Additionally, bacteriological examinations were performed on the swab samples from one carpal joint, the carpal bursa and one tarsal bursa. The calves’ blood work showed no pathological changes but revealed the notable absence of leukocytosis. In contrast, the microbiological results of the TTLs and the synovial swab samples confirmed the presence of several pathogenic agents responsible for BRD and septic arthritis. The TTLs contained *Trueperella pyogenes*, *Pasteurella multocida*, *Mannheimia haemolytica* and *E. coli*, whereas the synovial samples were positive for *Helcococcus ovis* and *Trueperella pyogenes*. Additional swab samples from TTLs showed positive results for *Mycoplasma spp.* (details of the microbiological examinations including antimicrobial resistance panels are provided in Tables 1 and 2).

**Table 1:** The detailed microbiological swab sample results of the tarsal bursa of calf 1 and the carpal bursa and joint of calf 2

Infectious Agent	Calf 1 (Right Tarsal Bursa)	Calf 2 (Right Carpal Bursa and Joint)
Helcococcus ovis	**+++**	**+++**
Staphylococcus hominis	-	**+**
Staphylococcus simulans	-	**+**
Trueperella pyogenes	-	**+++**
Acinetobacter wolfii	**+**	-
Corynebacterium sp.	-	-
E. coli	-	-
Staphylococcus vitulinus	**+**	-

**Table 2:** Antimicrobial resistance panels of the tested pathogens, including the antibiotics tested. The microdilution method was used, with a minimum inhibitory concentration provided in µg/mL

Antibiotic Substance	*Helcococcus ovis*	*Trueperella pyogenes*
Amoxicillin / Clavulanic acid	S ≤ 2 / 1	S ≤ 2 / 1
Ampicillin	S ≤ 0.25	S ≤ 0.25
Ceftiofur	S ≤ 0.125	S ≤ 0.125
Colistin	R > 2	R > 2
Cephalothin	S ≤ 1	S ≤ 1
Enrofloxacin	S = 0.25	S = 0.25
Erythromycin	S ≤ 0.125	S ≤ 0.125
Florfenicol	S ≤ 1	S ≤ 1
Gentamicin	I = 8	S = 2
Penicillin G	S ≤ 0.0625	S ≤ 0.0625
Spectinomycin	S ≤ 4	S ≤ 4
Trimethoprim / Sulfamethoxazol	S ≤ 0.25 / 4.75	S ≤ 0.25 / 4.75
Tetracyclin	S ≤ 0.125	S ≤ 0.125
Tiamulin	S ≤ 0.25	S ≤ 0.25
Tilmicosin	S ≤ 0.5	S ≤ 0.5
Tulathromycin	S ≤ 1	S ≤ 1

## Discussion and conclusion

The treatment of septic arthritis and tenosynovitis mainly entails joint lavages including antibiotic and anti-inflammatory approaches, as well as surgical techniques like arthrotomy or arthrodesis [22, 23]. Only hyperacute cases of septic arthritis may be treated conservatively, whereas surgery should be performed in chronic cases [22]. The decision to use one or the other surgical method to remove fibrin or detritus from the articular capsule depends on the nature of effusion [23]. Due to economic aspects, surgical techniques like arthroscopy or arthrotomy are reserved for animals with high genetic value and companion animals [22]. In this case report, two calves with chronic bursitis and arthritis were presented. The treatment of choice would have been a surgical approach with joint lavages via arthroscopy or arthrotomy [22, 23]. Due to the accumulation of septic arthritis cases at the dairy farm and financial limitations, a larger focus was placed on the prevention instead of treating these diseases. Therefore, the focus was shifted to the results obtained from the bacteriological examinations. In the calves with septic bursitis or arthritis, several bacteria, including *Staphylococcus spp.*, *Acinetobacter wolfii*, *Trueperella pyogenes* and *Helcococcus ovis*, were isolated from the synovial cavities. Interestingly, *Helcococcus ovis* was detected in calf 1, without a co-infection with the bacteria mentioned above.

*Helcococcus ovis* was first described in sheep in the late 1990 s [24]. Since then, this bacterium has been co-isolated from cattle suffering from different diseases such as valvular endocarditis [25], metritis [26] and associated with bovine abortion cases [17]. In human medicine, *Helcococcus ovis* was isolated from an artificial eye infection [27]. In all these reports, co-infections with *T. pyogenes*, *Streptococcus spp., Staphylococcus aureus* or *E. coli* were present. In contrast to this, the significance and importance of *Helcococcus spp*. as a primary animal pathogen has only been recognised in pigs recently [20]. The pathogenic potential of *Helcococcus ovis* has been described in several species, including ruminants and *Helcococcus spp.* was found in pure cultures of mainly the lungs and the uterus or vagina of cattle and sheep [20]. It would have been preferable to perform a necropsy on calf 2, in order to determine whether *Helcococcus ovis* was involved in the cause of the respiratory diseases of the calves at this dairy farm. However, the bacteriological examinations of the transtracheal lavages tested negative for *Helcococcus spp.*. Furthermore, the dairy cows should have been tested for *Helcococcus ovis* in association with the diseases mentioned initially. Although *Helcococcus ovis* is regarded as part of the normal postpartum intrauterine microbiome [28], its transfer to calves may have led to a higher infectious potential of this bacterium. Unfortunately, sampling of the cows was not possible, as the owner did not request the second herd health visit as suggested.

General recommendations to reduce the incidence of septic bursitis and arthritis, such as improving stable hygiene and immediately installing a separate box for calving, were made. At the time of the herd health visit, calves were born in the slatted floor cubicle housing system. It was presumed that the neonatal mucous membranes and the umbilical regions were contaminated with the microflora of the dairy cows. Passive transfer failure and ascending umbilical infections often lead to septic arthritis or polyarthritis [23]. Thus, an additional sample of first-day colostrum was examined with a digital Brix refractometer yielding 11 % Brix. Due to the fact that a Brix refractometer value of less than 20 % corresponds with low-quality colostrum with less than 50 mg IgG/mL [29], the farmer was advised to collect and store high-quality colostrum. The amount of colostrum administered to each calf within the first hours of birth was considered sufficient (4 L within the first 6–8 h).

Due to the owner’s financial limitations, the calves were not transferred to the clinic. Instead, he was advised to observe his animals closely and contact the local veterinarian at the first signs of disease. After adhering to most of these suggestions, the herds’ health improved significantly. Nevertheless, the presence of *Helcococcus ovis* and its potential role as a primary pathogen in septic bursitis and arthritis in calves should not be underestimated, especially as the bacterium is emerging as a primary pathogen in several species [20].

## Data Availability

The datasets used and/or analysed during the study are available from the corresponding author upon request.

## Abbreviations

BRD: Bovine respiratory disease
PCR: Polymerase chain reaction
TTL: Transtracheal lavage
